# A Functional Skincare Formulation Mixed With Retinyl Propionate, Hydroxypinacolone Retinoate, and Vitamin C on Antiaging and Whitening Han Women in Shanghai, China

**DOI:** 10.1111/jocd.16747

**Published:** 2025-03-03

**Authors:** Wei Fang, Jian Qiao, Fan Zhang, Min Chen, Qianjin Bian

**Affiliations:** ^1^ Department of Laser and Aesthetic Medicine, Shanghai Ninth People's Hospital Shanghai JiaoTong University School of Medicine Shanghai China; ^2^ Zhan Qiu (Shanghai) Biotechnology Co. Ltd. Shanghai People's Republic of China; ^3^ Department of Dermatology Changzheng Hospital Shanghai People's Republic of China

**Keywords:** aging face, creams, retinyl propionate, whitening agents

## Abstract

**Background:**

Human skin is remodeled as a result of aging. Retinoids (or retinol derivatives) can intervene in this process, which can be optimally formulated and concentrated by providing maximum antiaging effects with minimal irritation.

**Aims:**

To determine the optimal ratio of two retinol derivatives hydroxypinacolone retinoate (HPR), retinol propionate (RP), and vitamin C (VitC) in dermal remodeling and preventing skin aging, and to investigate their synergistic antiaging and whitening effects in vitro and in vivo.

**Methods:**

An in vitro model of human skin fibroblast (HSF) was established to evaluate the cellular viability of VitC and/or RP/HPR treatment. We quantified the expression of genes associated with antiaging and retinol receptors in HSFs treated with VitC and/or RP/HPR. The research was conducted on 120 volunteer women to quantify the antiaging efficacy of functional skincare formulation.

**Results:**

A combination of VitC and RP/HPR at a weight ratio of 40:3 achieved the optimal and safe antiaging performance in vitro. Transcription of aging‐associated genes and the release of elastin and senescence‐associated secretory phenotypes (SASPs) increased significantly after UV irradiation, whereas VitC and RP/HPR cotreatment can inhibit this increase. The synergistic stimulation of VitC and RP/HPR cotreatment showed higher efficacy in the recovery ability of skin injury.

**Conclusions:**

Our results indicated the advanced skin antiaging and whitening effects of VitC and RP/HPR cotreatment in vitro and in vivo. A combination of VitC and RP/HPR is a potent strategy to postpone skin aging and improve skin whitening for middle‐aged women.

**Trial Registration:**

ClinicalTrials.gov identifier: 2020‐LS‐023

## 1 Introduction

As the barrier of the human body, skin comprising the epidermis, dermis, and subcutaneous tissue is exposed to stimuli that influence its morphology and function [1, 2]. Generally, age‐related skin changes are mainly attributed to the accumulation of senescent cells, which are associated with progressive atrophy of the dermis, including wrinkles and reduced skin elasticity [2]. Collagen synthesis is reduced in older skin, whereas its degradation is increased, leading to a disorganized extracellular matrix (ECM) [3].

Retinol propionate (RP) is a vitamin‐A derivative, which has an antiaging effect and skin texture improvement [4, 5]. RP can promote collagen synthesis, reduce the occurrence of wrinkles and fine lines, improve the pigmentation and roughness of skin [6, 7]. Hydroxypinacolone retinoate (HPR) is an ester of retinoic acid, which has antioxidant, antiaging, skin‐whitening effects [1]. HPR can also reduce skin pigmentation, improve uneven skin tone, and reduce adverse reactions such as dry skin [4, 8]. Vitamin C (VitC) is a powerful antioxidant, it can promote collagen synthesis, and improve the elasticity and tightness of the skin [1, 9, 10].

However, the effect of a combination of “RP, HPR, and VitC” has not been studied on the antiaging and whitening of facial skin. Thus, we evaluated the effect of the antiaging, whitening, and radiance of skin using a combination of “RP/HPR and VitC” in vitro and in vivo. In addition, the efficacy of “RP/HPR and VitC” on antiging, whitening, and expression of genes associated with cell senescence was also detected using an in vitro human skin fibroblast (HSF) model. Moreover, the optimal weight ratio between RP/HPR and VitC was identified in our study.

## 2 Materials and Methods

### 2.1 Human Volunteer Study

According to State Food and Drug Administration, cosmetic safety technical standards set by the Chinese government and a clinical trial registration number “2020‐LS‐023” by Changzheng Hospital, 120 of healthy Han women volunteers (25–40 years old) were recruited randomly and divided randomly into four groups of 30. Women were given daily care of facial skin for 28 days (twice a day) with: skincare serum; skincare serum containing VitC; skincare serum containing RP and HPR (weight ratio of RP: HPR = 2:1); skincare serum containing VitC and RP/HPR (weight ratio of VitC: RP/HPR = 40:3). A single‐center, open, self‐control cohort study was used to investigate the antiaging and whitening parameters of facial skin in the groups stated above using the Visia skin analyzer, Visia‐CR (Courage + Khazaka electronicGmbH), and Primos‐CR (CANFIELD) on days 0, 14, and 28. The technician then evaluated the grades of the fine grain area, crow's feet and nasolabial folds [11, 12]. The inclusive and exclusive criteria of human volunteer study were shown in Appendix S1.

### 2.2 Cell Viability

HSF suspension was cultured in a 96‐well plate for 24 h. Blank and control groups were set up simultaneously. Complete culture medium was used, vitC (0, 17.6, 88, 440, 2200 μg/mL) and RP/HPR (0, 1.32, 6.6, 33, 165 μg/mL) were added to each well for six repeat experiments, and incubate for 24 h. The cell culture supernatant was removed, and Cell Counting Kit‐8 solution was added to each well, followed by incubation for 2 h. Determination of absorbance at 450 nm was achieved using an enzyme‐labeling instrument.

### 2.3 Skin‐Tissue Experiment In Vitro

After mouse‐derived skin tissue had been cultured for 2 days, five groups were set up: control, UV, “UV + VitC”, “UV + RP/HPR” and “UV + VitC+RP/HPR”. Doses of ultraviolet (UV)‐A (UVA) (30 J/cm^2^) and UVB (50 mJ/cm^2^) were irradiated continuously for 4 days. Tissue sections were made for staining (hematoxylin and eosin (H&E), Masson, immunofluorescence).

### 2.4 Cell Experiment In Vitro

HSFs were inoculated and cultured in six‐well plates for 24 h. Complete medium was used to set up five groups: control, UV, “UV + VitC”, “UV + RP/HPR” and “UV + VitC+RP/HPR”. The dose of UV was 50 mJ/cm^2^. Then, groups of VitC, “RP/HPR” and “VitC+RP/HPR” were given, respectively, for 24 h, followed by staining (β‐galactosidase and Sirius Red). HSFs were taken to make a single‐cell suspension, seeded on a six‐well plate, and cultured in medium containing 10% fetal bovine serum for 24 h. After centrifugation, the supernatant was discarded. Total cellular RNA was extracted from each well. qPCR was used to quantify mRNA expression of P21, P16, COL1A1, COL4A1, COL6A1, and CRABP2. ELISAs were used to quantify protein expression of elastin, CCL‐5, CXCL‐10, PAI‐1, TNF‐α, MMP‐3, and MCP‐1. Western blotting was used to measure protein expression of CRABP2.

### 2.5 Statistical Analyses

SPSS 23 (IBM, Armonk, NY, USA) was used to analyze data. Measurement data with a normal distribution are expressed as the mean ± standard error. The paired *t*‐test was employed for the comparison of data between the two groups. Rank sum tests were used for comparison of grades before and after treatment. The independent sample *t*‐test or rank sum test was used for comparison between the treatment groups and control group. *p* = 0.05 (two‐tailed) was considered significant.

## 3 Results

### 3.1 Human Volunteer Study

#### 3.1.1 Comparison of Skin Elasticity, Compactness, and Skin Gloss

The elasticity and firmness of the skin are crucial indicators for assessing youthfulness of skin, and influenced by the content and quality of collagen, elastin fibers, skin‐moisture content, muscle tension, and genetic factors. We evaluated the firmness and elasticity of the skin by measuring values of F4 and R2. The magnitude of F4 is negatively correlated with skin firmness. The magnitude of R2 is positively correlated with skin elasticity. The individual type angle (ITA) was calculated by measuring L*, a*, and b* values using a skin chromometer, which was used to evaluate the smoothness and glossiness of skin (Table 1). Compared with the control group, therapy with “RP/HPR + VitC” for 14 days or 28 days led to a significant improvement in F4, R2, ITA, L*, a*, and b* values (Appendix S2).

**TABLE 1:** Comparison of Cutometer test value and Visia‐CR value between four groups of subjects.

Group	Time	Cutometer test		Visia‐CR value			
		R2 value	F4 value	L* value	a* value	b* value	ITA° value
Group A	Day 0	0.54 ± 0.01	2.78 ± 0.05	69.14 ± 0.56	12.32 ± 0.31	20.54 ± 0.56	43.12 ± 1.46
	Day 14	0.57 ± 0.01^###^	2.66 ± 0.05^##^	69.16 ± 0.52	12.24 ± 0.32	20.52 ± 0.55	43.20 ± 1.36
	Day 28	0.58 ± 0.01^###^	2.56 ± 0.05^###^	69.24 ± 0.46	12.23 ± 0.28	20.51 ± 0.49	43.20 ± 1.23
Group B	Day 0	0.55 ± 0.01	2.75 ± 0.07	70.32 ± 0.67	11.17 ± 0.40	18.33 ± 0.57	47.78 ± 1.53
	Day 14	0.58 ± 0.01^###^	2.56 ± 0.07^###^	71.02 ± 0.57	10.86 ± 0.37	18.55 ± 0.55	48.51 ± 1.39
	Day 28	0.59 ± 0.01^###^	2.51 ± 0.07^###^	70.88 ± 0.67	10.99 ± 0.42	18.33 ± 0.55	48.54 ± 1.49
Group C	Day 0	0.53 ± 0.01	2.83 ± 0.07	69.55 ± 0.66	11.73 ± 0.45	18.60 ± 0.59	46.33 ± 1.66
	Day 14	0.58 ± 0.01^###^	2.66 ± 0.07^#^	70.35 ± 0.62^##^	11.65 ± 0.37	18.59 ± 0.57	47.53 ± 1.46^#^
	Day 28	0.60 ± 0.01^###^	2.55 ± 0.06^###^	70.46 ± 0.63^#^	11.64 ± 0.39	18.51 ± 0.64	47.85 ± 1.55^#^
Group D	Day 0	0.52 ± 0.01	3.03 ± 0.08	69.03 ± 0.55	12.28 ± 0.35	18.55 ± 0.56	45.74 ± 1.42
	Day 14	0.58 ± 0.01^###^	2.70 ± 0.07^###^	69.82 ± 0.57^##^	12.10 ± 0.37	18.26 ± 0.61	47.39 ± 1.50^###^
	Day 28	0.60 ± 0.01^###^	2.57 ± 0.08^###^	70.13 ± 0.58^###^	11.76 ± 0.37^#^	18.10 ± 0.54	47.98 ± 1.41^###^

*Note:* Group A: skincare serum; Group B: skincare serum containing VitC; group C: skincare serum containing RP/HPR; Group D: skincare serum containing RP/HPR and VitC.

^#^ *p* < 0.05.

^##^ *p* < 0.005.

^###^ *p* < 0.001.

#### 3.1.2 Comparison of Facial Wrinkles

We evaluated the changes of facial wrinkles by comparing the fine grain area and lateral canthal lines (“crow's feet”) before and after treatment in the four groups. The number, area, and volume of nasolabial folds were compared before and after treatment (Table 2). Compared with the control group (Group A), therapy with “RP/HPR + VitC” for 14 days or 28 days led to a significant improvement in the facial wrinkles. As for the evaluation by technician, the valuation of facial wrinkles was based on changes from baseline was conducted at 14 and 28 days. Compared with baseline, Group D noticed a significant improvement in the fine grain area and crow's feet at 14 days and 28 days. Group D reported no significant improvement in nasolabial folds until 28 days (Table 3).

**TABLE 2:** Comparison of nasolabial fold parameters and facial wrinkles parameters between four groups of subjects.

Group	Time	Nasolabial folds			Facial wrinkles	
		Number of nasolabial folds	Area of nasolabial folds	Volume of nasolabial folds	Ratio of fine grain area	Ratio of crow's feet
Group A	Day 0	50.33 ± 2.75	16.83 ± 0.70	0.87 ± 0.06	0.05 ± 0.01	0.02 ± 0.00
	Day 14	46.40 ± 2.77^###^	15.20 ± 0.77	0.78 ± 0.06	0.05 ± 0.01	0.02 ± 0.00
	Day 28	45.97 ± 2.53^###^	14.89 ± 0.77	0.77 ± 0.06	0.05 ± 0.01	0.02 ± 0.00
Group B	Day 0	54.80 ± 2.62	17.46 ± 0.65	0.83 ± 0.04	0.05 ± 0.00	0.02 ± 0.00
	Day 14	48.00 ± 2.13^###^	14.95 ± 0.67	0.69 ± 0.05	0.04 ± 0.00	0.02 ± 0.00
	Day 28	47.37 ± 1.88^###^	14.20 ± 0.69	0.67 ± 0.04	0.04 ± 0.00	0.02 ± 0.00
Group C	Day 0	52.63 ± 3.48	16.16 ± 0.81	0.81 ± 0.06	0.07 ± 0.01	0.03 ± 0.00
	Day 14	45.40 ± 2.81^###^	13.80 ± 0.80	0.68 ± 0.07	0.07 ± 0.01	0.03 ± 0.00
	Day 28	43.80 ± 2.64^###^	13.12 ± 0.81	0.65 ± 0.06	0.06 ± 0.01	0.03 ± 0.00
Group D	Day 0	52.07 ± 1.78	16.31 ± 0.68	0.77 ± 0.05	0.05 ± 0.01	0.02 ± 0.00
	Day 14	45.17 ± 1.79^###^	13.80 ± 0.70	0.63 ± 0.05	0.04 ± 0.01	0.02 ± 0.00
	Day 28	43.17 ± 2.01^###^	12.84 ± 0.73	0.59 ± 0.04	0.04 ± 0.01	0.02 ± 0.00

*Note:* Group A: skincare serum; Group B: skincare serum containing VitC; group C: skincare serum containing RP/HPR; Group D: skincare serum containing RP/HPR and VitC.

^#^ *p* < 0.05.

^##^ *p* < 0.005.

^###^ *p* < 0.001.

**TABLE 3:** The grades of facial wrinkles evaluated by technician before and after using matrix essence containing RP/HPR and VitC.

Group	Time	Nasolabial folds	Fine grain area	Crow's feet
Group A	Day 0	2.70 ± 0.13	3.60 ± 0.13	3.23 ± 0.14
	Day 14	2.63 ± 0.12	3.47 ± 0.12^#^	3.03 ± 0.14^#^
	Day 28	2.53 ± 0.10^#^	3.40 ± 0.13^#^	2.93 ± 0.14^##^
Group B	Day 0	2.30 ± 0.12	3.20 ± 0.18	2.73 ± 0.14
	Day 14	2.23 ± 0.12	2.93 ± 0.17^##^	2.53 ± 0.10^#^
	Day 28	2.10 ± 0.11^#^	2.87 ± 0.17^##^	2.40 ± 0.12^##^
Group C	Day 0	2.43 ± 0.10	3.27 ± 0.14	2.97 ± 0.13
	Day 14	2.33 ± 0.10	3.00 ± 0.14^##^	2.70 ± 0.12^##^
	Day 28	2.23 ± 0.09^#^	2.93 ± 0.14^##^	2.57 ± 0.14^##^
Group D	Day 0	2.20 ± 0.12	4.03 ± 0.18	3.13 ± 0.16
	Day 14	2.10 ± 0.11	3.63 ± 0.16^###^	2.87 ± 0.13^##^
	Day 28	2.00 ± 0.11^#^	3.53 ± 0.16^###^	2.63 ± 0.13^###^

*Note:* Group A: skincare serum; Group B: skincare serum containing VitC; group C: skincare serum containing RP/HPR; Group D: skincare serum containing RP/HPR and VitC.

^#^ *p* < 0.05.

^##^ *p* < 0.005.

^###^ *p* < 0.001.

### 3.2 Skin Complexion

**FIGURE 1:**
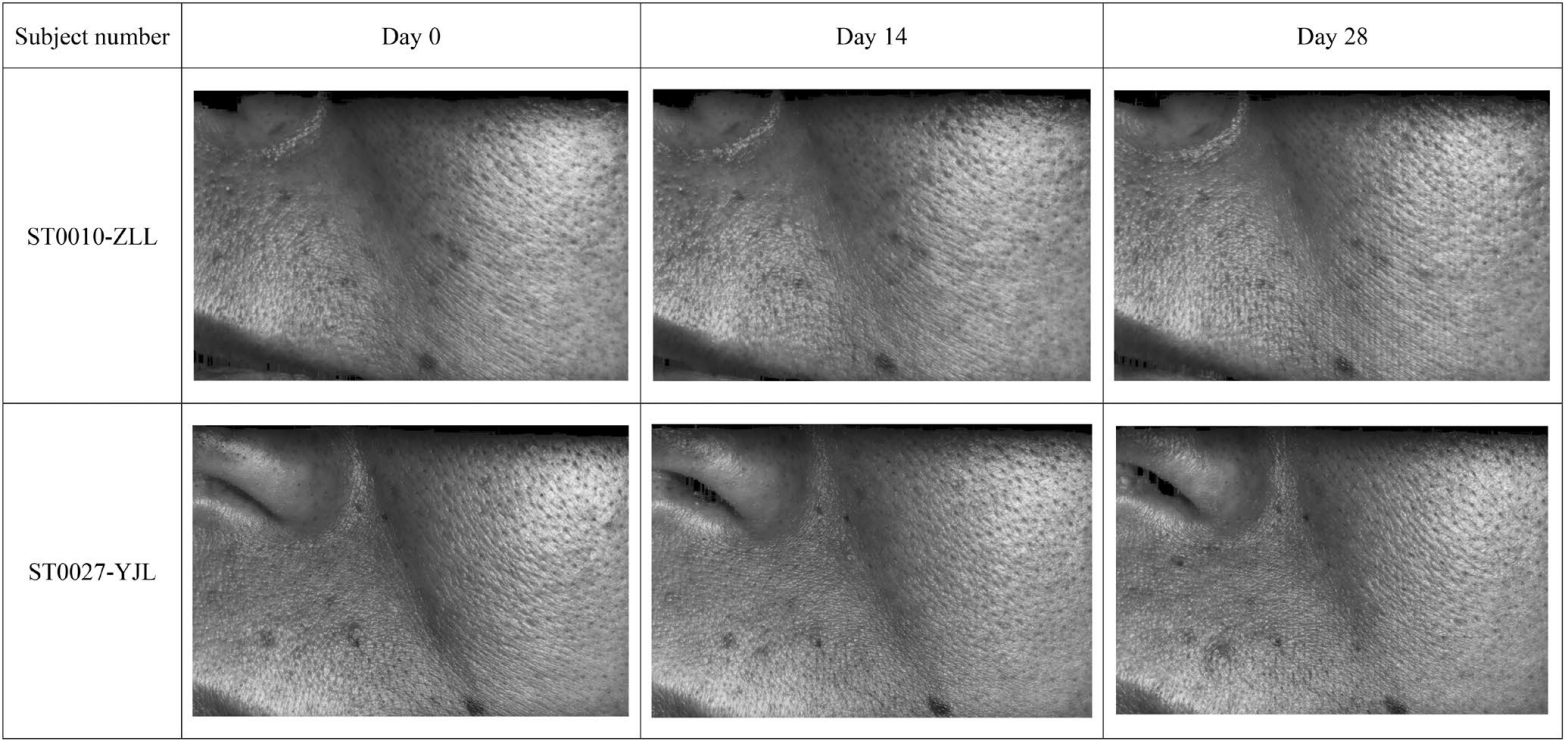
Multidimensional imaging comparison of the facial skin of representative subjects before and after using serum containing RP/HPR and VitC.

**FIGURE 2:**
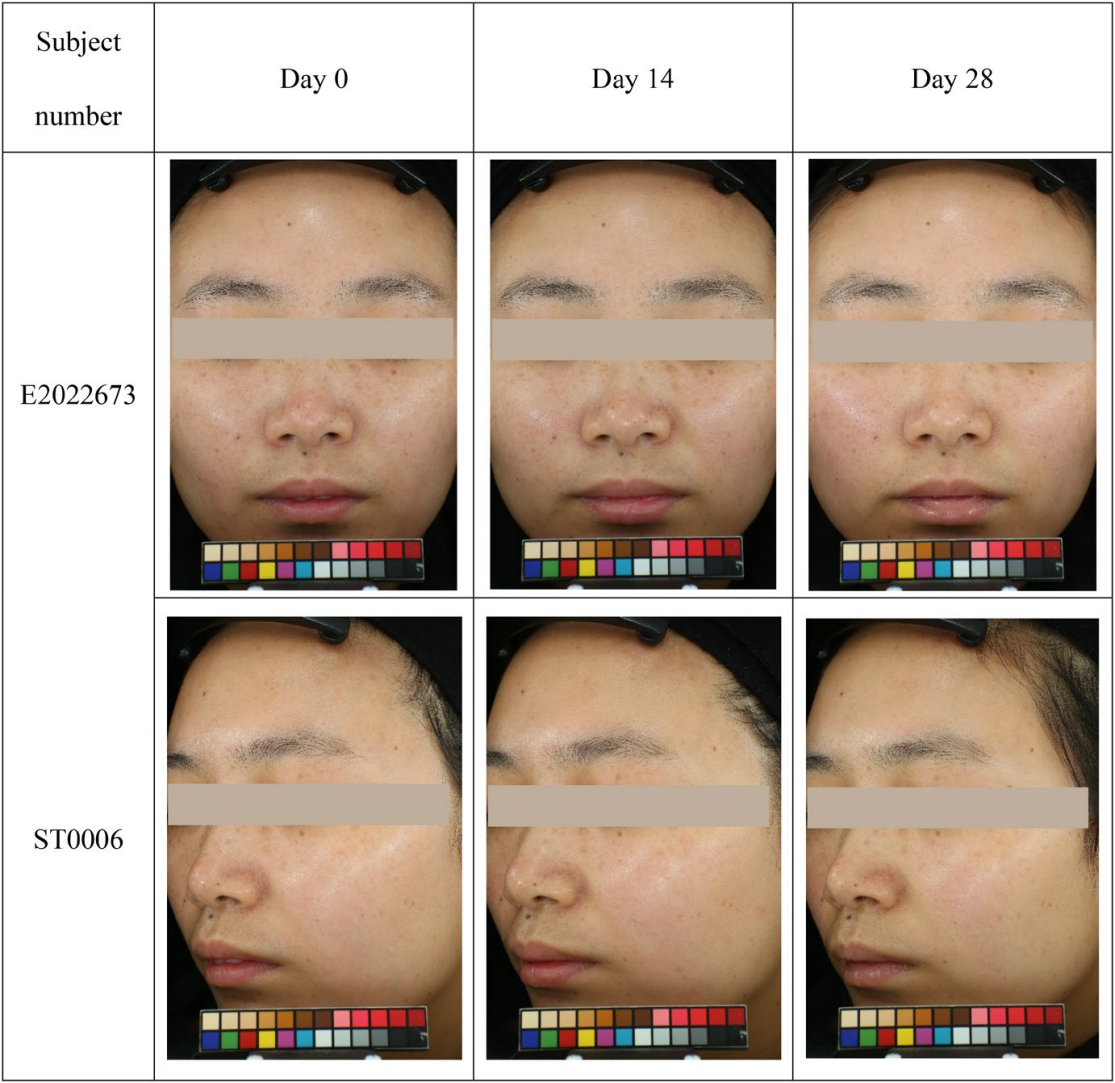
Comparison of facial wrinkle improvement in representative subjects before and after using skincare serum containing RP/HPR and VitC.

As indicated by Visia‐CR and Primos‐CR (Figures 1 and 2), the overall improvement in skin complexion included: pore improvement; reduction of Crow's feet, marionette, and nasolabial lines; improvement in the texture and smoothness of the skin; enhancement of the tone and radiance of the skin.

### 3.3 Cell Viability

**FIGURE 3:**
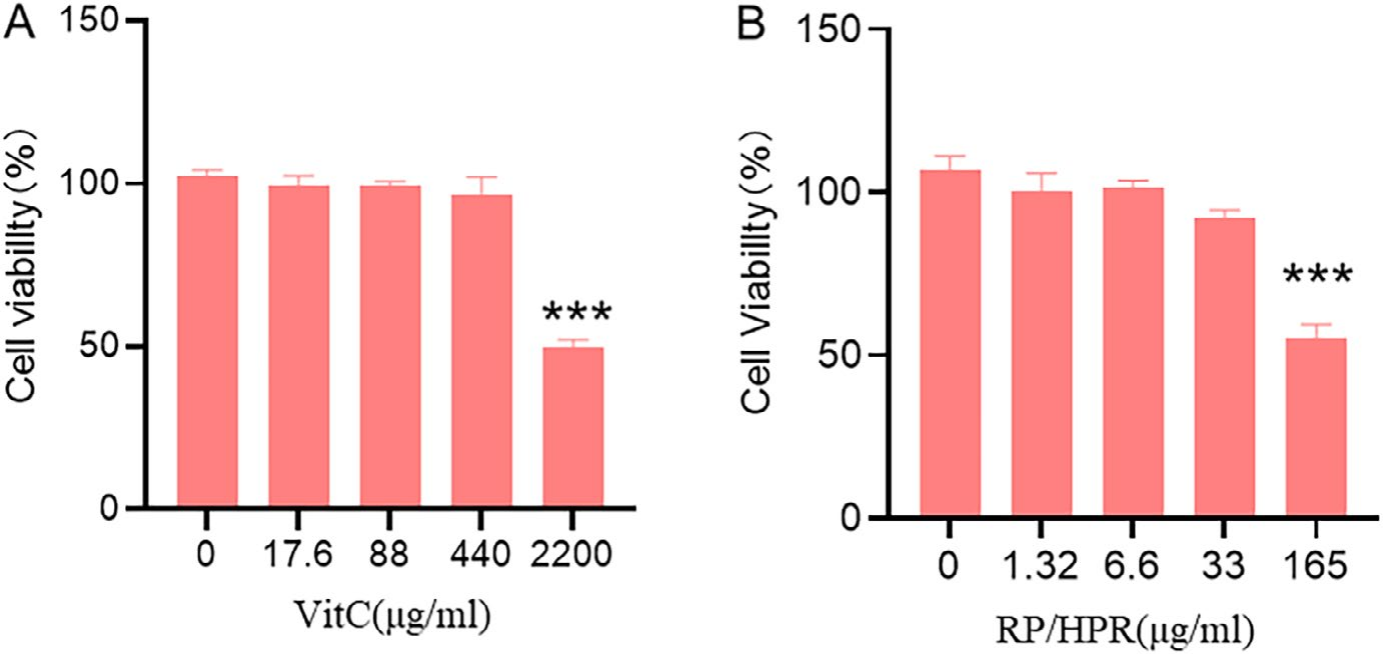
Cell viability of HSF cells at different concentrations VitC (A) or RP/HPR (B) treatment assessed at 24 h. ^***^ *p* < 0.001.

HSFs were incubated with RP/HPR or VitC at different concentrations for 24 h, and the cell‐viability results are shown in Figure 3. VitC at 2200 μg/mL (Figure 3A) and RP/HPR at 165 μg/mL (Figure 3B) showed toxicity to HSFs compared with the control treatment. Negligible toxicity was observed if the VitC concentration was ≤ 440 μg/mL and RP/HPR concentration was ≤ 33 μg/mL.

### 3.4 Skin‐Tissue Experiment In Vitro

**FIGURE 4:**
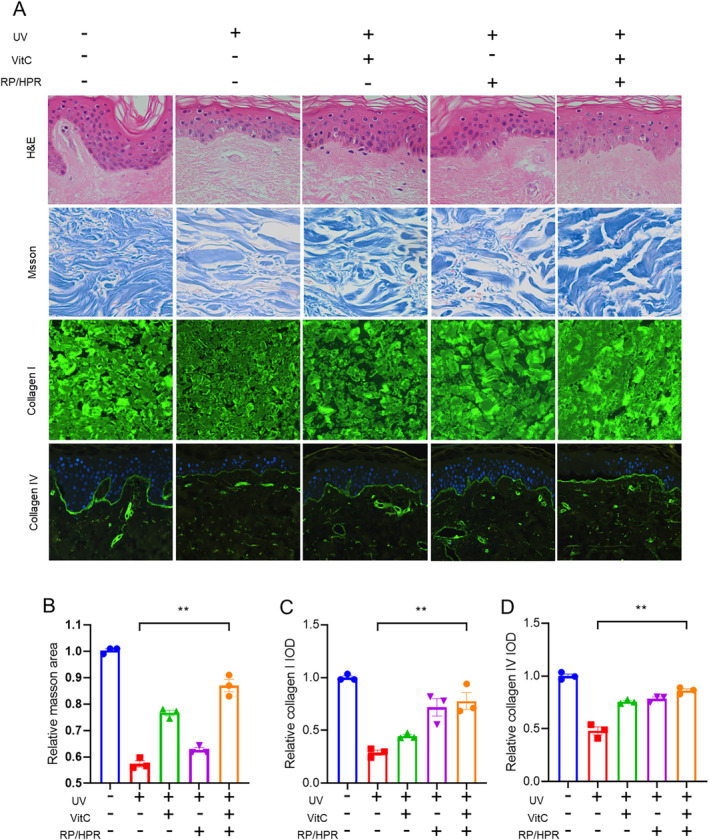
Skin tissue treated with RP/HPR and/or VitC experiment in vitro. **p < 0.005.

After 4 days of UV irradiation, isolated skin tissue was treated with RP/HPR and/or VitC, followed by staining (H&E, Masson, immunofluorescence) (Figure 4). H&E staining showed that UV irradiation could cause skin injury, reduce the thickness of the epidermis, as well as break and degrade collagen fibers. Besides, expression of COL‐I and COL‐IV in skin was decreased significantly. Therapy with VitC or RP/HPR could repair the skin damage caused by UV irradiation, increase the thickness of the epidermis, and restore expression of COL‐I and COL‐IV. Compared with single treatment with RP/HPR or VitC, the recovery ability of skin injury and fragmentation of collagen fibers of synergistic stimulation by RP/HPR and VitC cotreatment was improved significantly, as well as decreased expression of COL‐I and COL‐IV induced by UV irradiation.

### 3.5 Cell Experiment In Vitro

**FIGURE 5:**
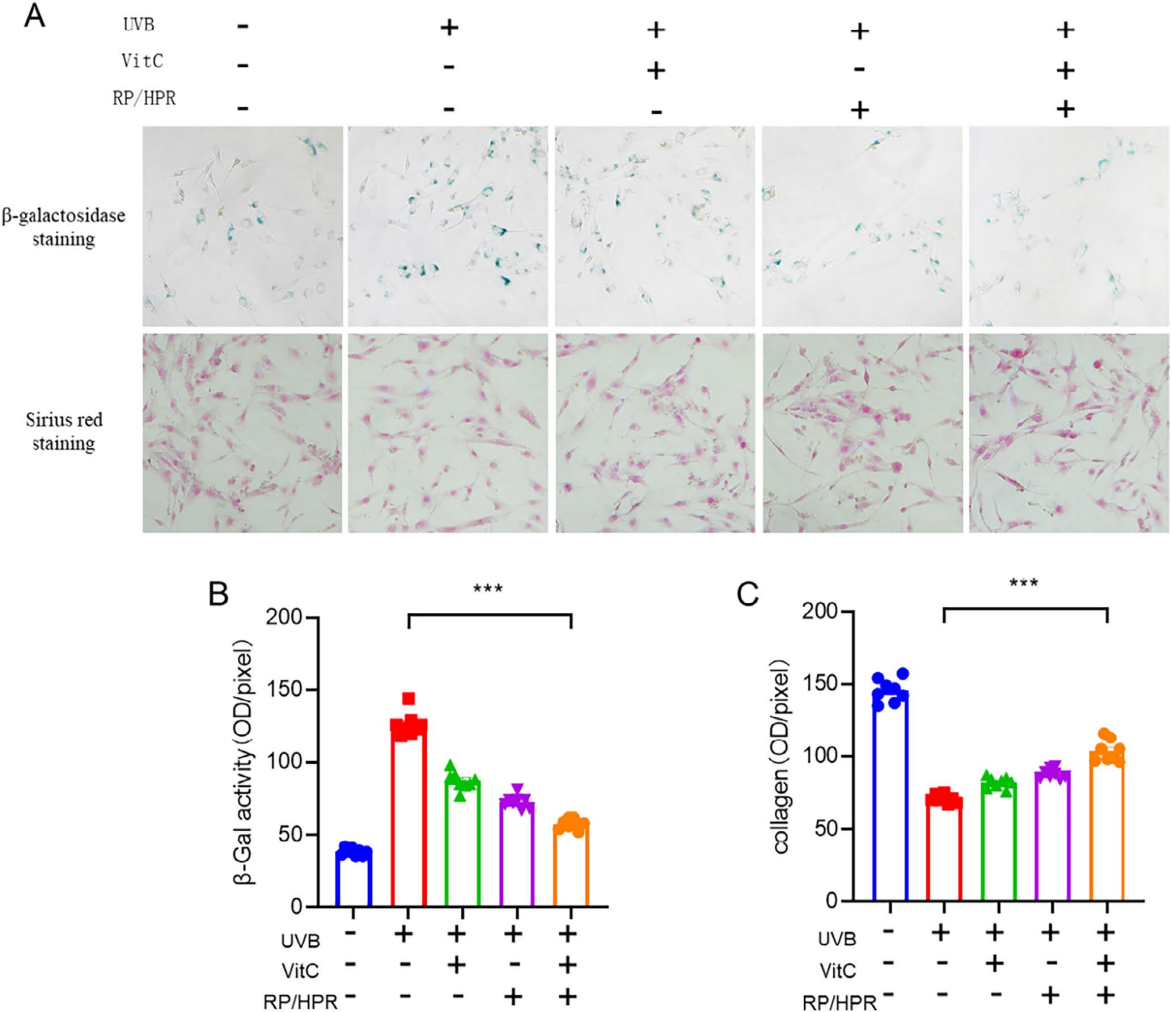
Cell treated with RP/HPR and/or VitC experiment in vitro. ***p < 0.001.

HSFs were treated with RP/HPR and/or VitC after UV irradiation, followed by staining (β‐galactosidase and Sirius Red) (Figure 5). β‐galactosidase activity increased significantly and COL‐I expression decreased significantly after UV irradiation. Compared with treatment with RP/HPR alone or VitC alone, the synergistic stimulation elicited by RP/HPR and VitC cotreatment showed greater efficacy.

**FIGURE 6:**
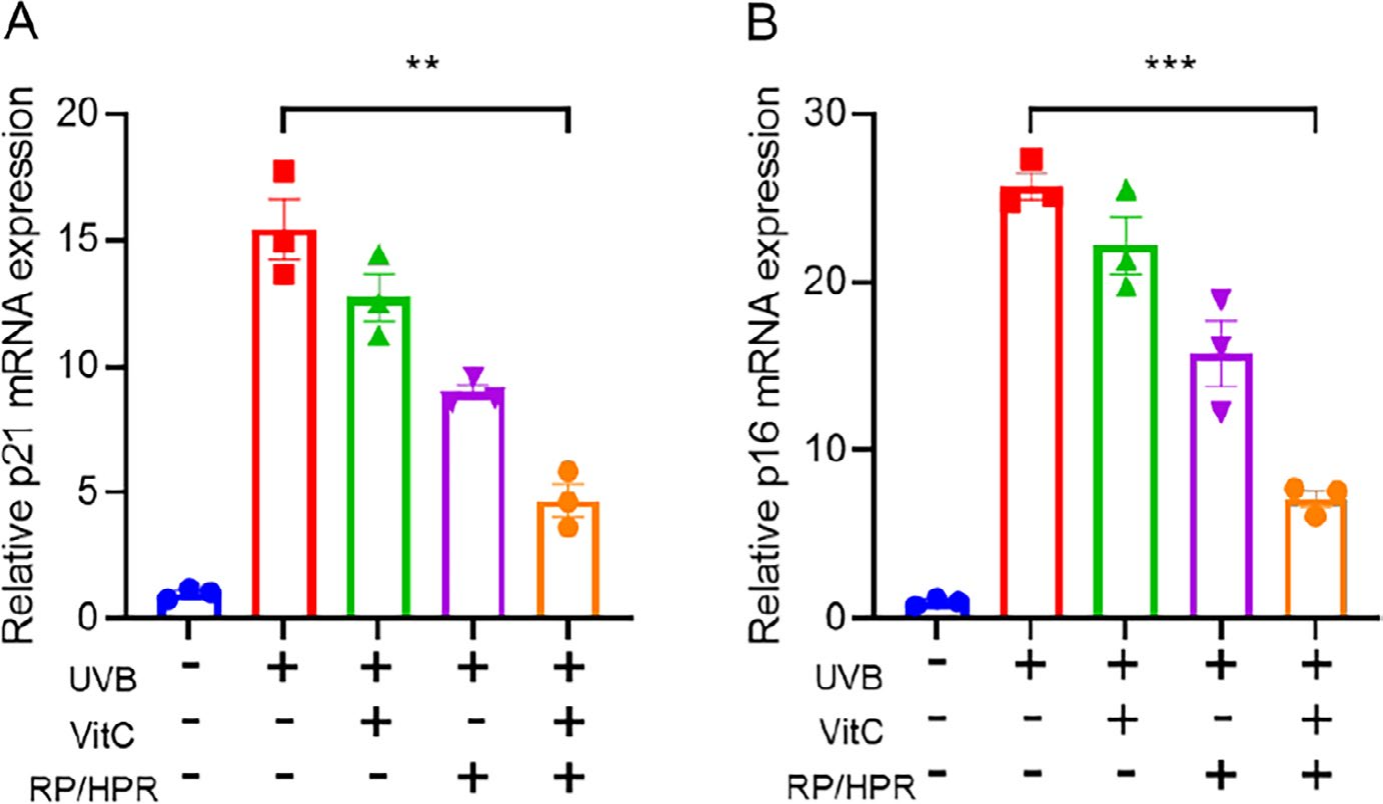
The effect of vitamin C and/or RP/HPR on the expression of p21 (A) and p16 (B) mRNA related to UV‐induced cell senescence. **p < 0.005; ***p < 0.001.

P21 and P16 are genes are related directly to cell senescence. We detected the effect of VitC and/or RP/HPR on the expression of p21 mRNA and p16 mRNA related to UV‐induced cell senescence (Figure 6A,B). Transcription of p21 and p16 increased significantly after UV irradiation. Compared with RP/HPR monotherapy or VitC monotherapy, RP/HPR and VitC cotreatment inhibited the increase in transcription of p21 and p16 induced by UV irradiation.

**FIGURE 7:**
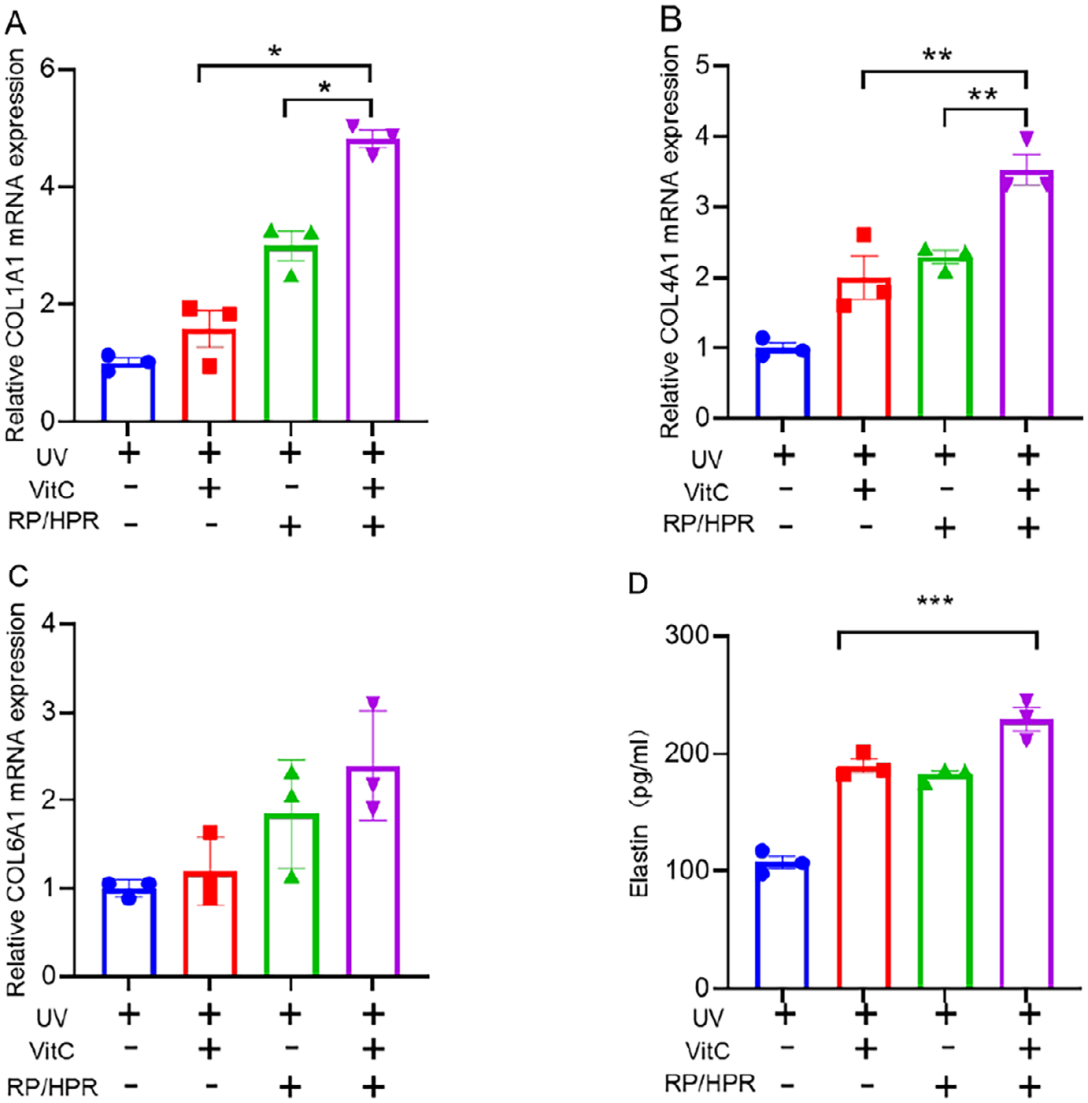
The effect of vitamin C and/or RP/HPR on the expression of collagen (A–C) and Elastin (D) related to UV‐induced HSF cell senescence. *p < 0.05; **p < 0.005; ***p < 0.001.

Cell senescence is also related directly to the amount of collagen expressed by fibroblasts. The effect of VitC and/or RP/HPR on the expression of collagen related to UV‐induced HSF senescence was detected. Compared with RP/HPR monotherapy or VitC monotherapy, RP/HPR and VitC cotreatment could promote the gene expression of COL‐I and COL‐IV significantly (Figure 7A,B). However, compared with the control group, the change in COL‐VI expression was not significant (Figure 7C). Besides, RP/HPR and VitC cotreatment could increase elastin release (Figure 7D).

**FIGURE 8:**
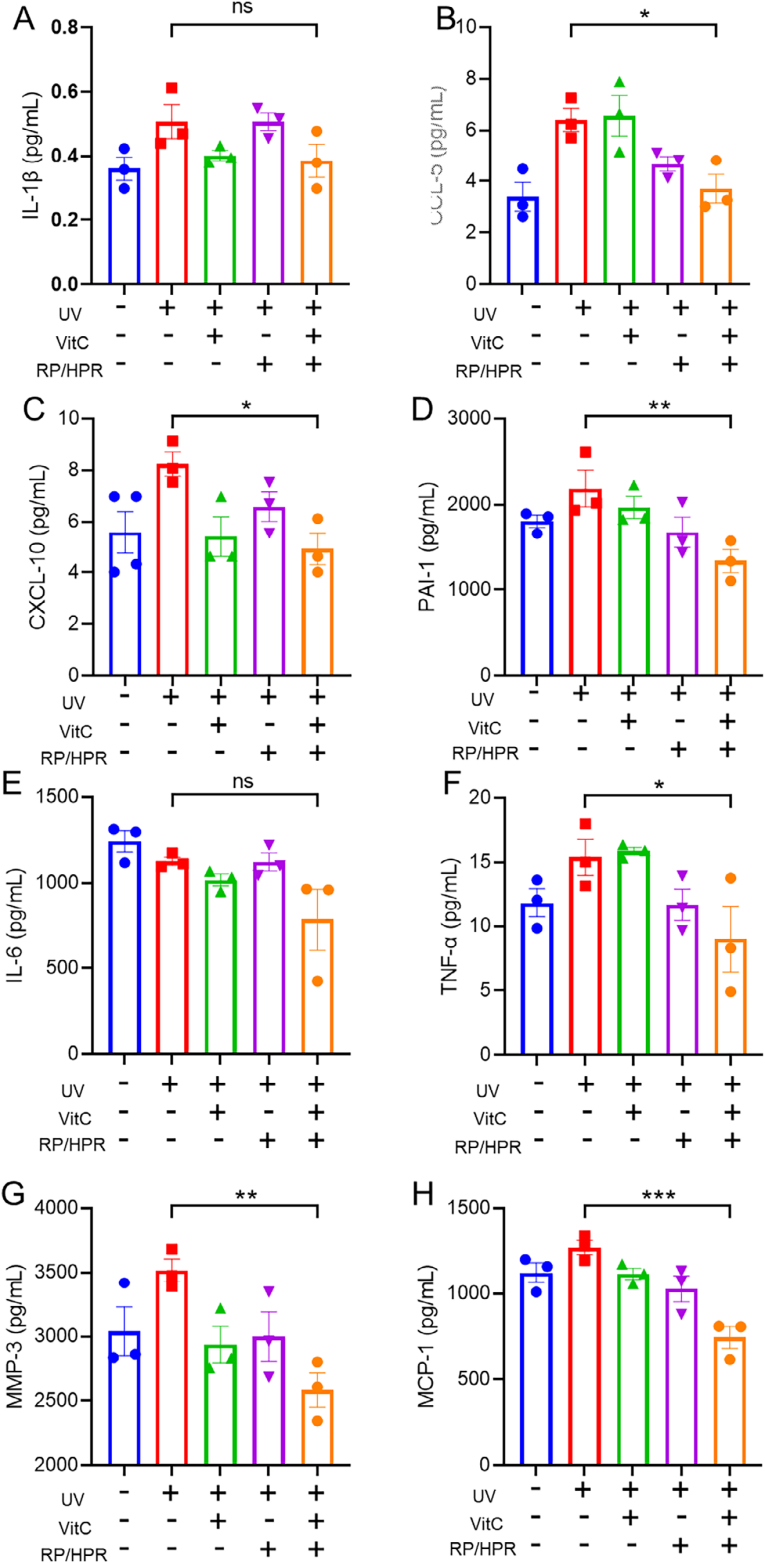
The effect of vitamin C and/or RP/HPR on the release of SASP related to UV‐induced HSF cell senescence. *p < 0.05; **p < 0.005; ***p < 0.001.

Cell senescence is also closely related to “inflammageing”. Cells secrete a series of proinflammatory cytokines, chemokines, growth factors, and proteases upon senescence initiation, which are referred collectively to as “senescence‐associated secretory phenotypes” (SASPs). The effect of VitC and/or RP/HPR on SASPs was detected using ELISAs. RP/HPR and VitC cotreatment could inhibit the release of CCL‐5, CXCL‐10, PAI‐1, TNF‐α, MMP‐3, and MCP‐1 induced by UV irradiation (Figure 8).

**FIGURE 9:**
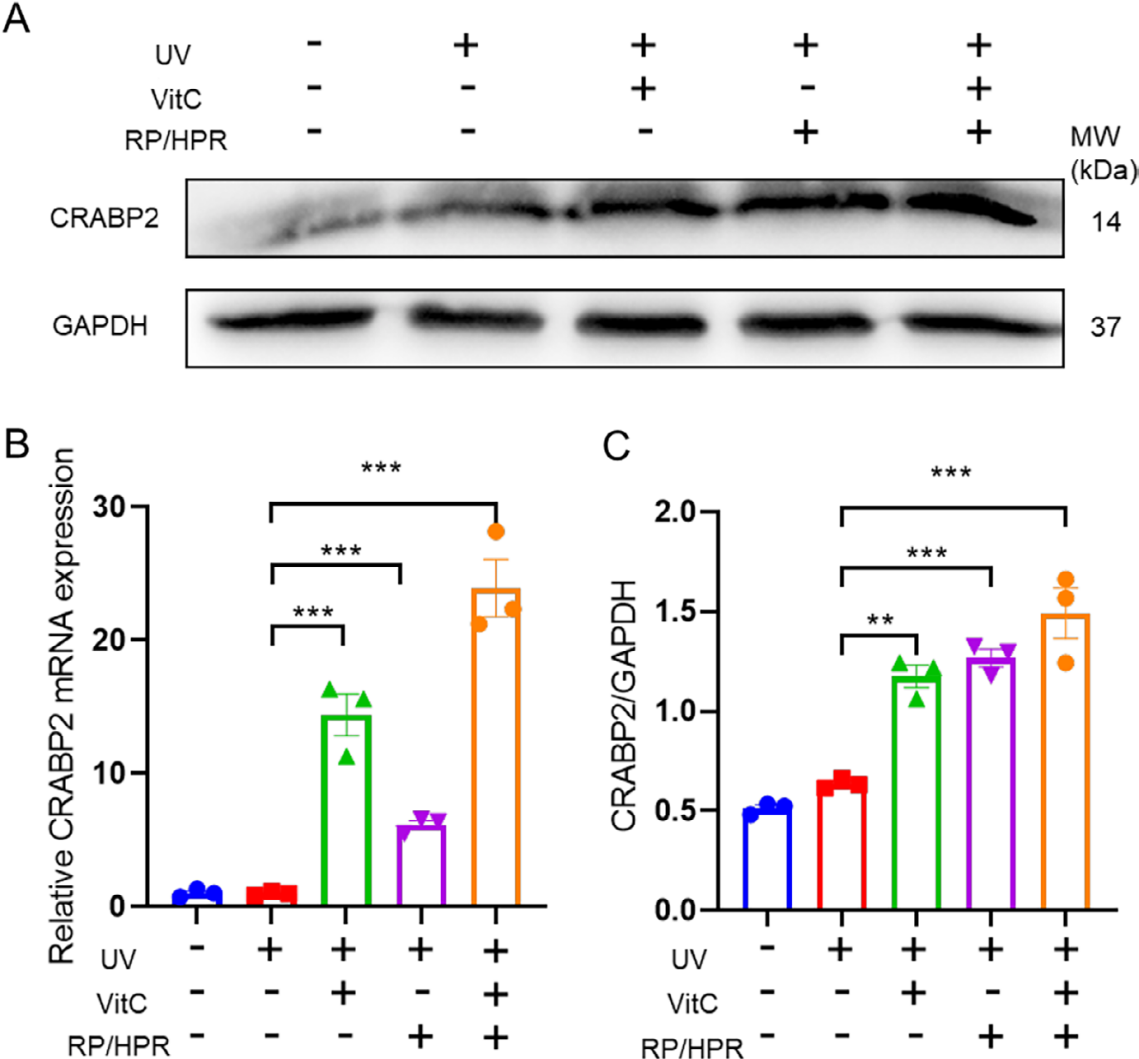
The effect of vitC and/or RP/HPR on the expression of CRABP2. **p < 0.005; ***p < 0.001.

It has been reported that HPR can be metabolized to retinoic acid in vivo and that CRABP2 plays an important part in mediating the transport of retinoic acid [7]. Therefore, we detected the effect of VitC and/or RP/HPR on the expression of CRABP2 by qPCR and western blotting. RP/HPR and VitC cotreatment could promote CRABP2 expression in HSFs induced by UV irradiation (Figure 9).

## 4 Discussion

Dermatological and epidermal homeostasis are impacted by retinoids [13]. In addition to regulating the biosynthesis of ECM components and maintaining dermal thickness, retinoids normalize the proliferation and differentiation of cells [14]. The dermal ECM is composed primarily of COL‐I and COL‐III, whereas COL‐IV collagen is present primarily in the basement membrane of the skin [15]. Aging skin does not have abundant collagen fibrils that are tightly packed and well‐organized, and collagen fibrils are fragmented and distributed coarsely [16].

Senescent cells exhibit dramatic changes in gene expression, chromatin organization, and expression of senescence‐associated β‐galactosidase [17, 18]. An increase in the activity of lysosomal β‐galactosidase in senescent cells is derived from lysosomal b‐D‐galactosidase. This behavior of β‐galactosidase is due to an increase in the number and size of lysosomes and, consequently, increased lysosome content in senescent cells [19].

In humans, genotoxic stress induces several factors associated with inflammation and malignancy to be secreted by cells, resulting in their senescence [20]. Several days are needed for SASPs to develop, and only after sufficient DNA damage has occurred to trigger senescence. After genotoxic stress, SASPs are also detected in epithelial tumor cells and normal fibroblasts [21]. It has been shown that ectopic expression of p16 and p21, induces senescence without a SASP [22]. p16 expression correlates directly with chronological aging of human skin in vivo, making it an effective biomarker of human aging [23]. Developmental senescence is a short‐term “programmed” type of cellular senescence that occurs during mammalian embryonic development, and it is accompanied by upregulation of p21 expression, which also is a valuable marker of senescence [24]. In addition to being induced during transient arrest of the cell cycle, it is also induced in response to DNA damage, and it should be used only in conjunction with other markers to indicate senescence [25].

Due to its higher chemical stability and greater safety profile, vitamin A ester has become a popular alternative to retinol and retinoic acid [4]. Recently, HPR has been shown to bind directly to retinoic acid receptors without undergoing biological transformation to retinoic acid [5]. Also, it is more stable and causes less skin irritation than the well‐known analog of vitamin A ester: RP [4]. VitC is a popular ingredient in over‐the‐counter “cosmeceuticals” due to its numerous biological functions, including the treatment of UV damage, improvement of discoloration, and stimulation of collagen growth [26].

Moreover, in the skin‐tissue experiment in vitro, VitC/HPR can repair the skin damage caused by UV irradiation by increasing the thickness of the epidermis and restoring the expression of COL‐I and COL‐IV (Figure 4). Hence, VitC and RP/HPR cotreatment elicited excellent antiaging and whitening functions, such results converge with previous studies [1]. Similar results were observed in the HSF aging model induced by UV irradiation (Figure 5). VitC monotherapy and RP/HPR monotherapy could slow‐down the increase of β‐galactosidase activity and decrease COL‐I expression caused by aging. It has been reported that the expression of CRBP is upregulated by both retinoic acid and retinol in human skin [27]. In our study, more importantly, the effect of VitC and RP/HPR cotreatment was significantly strong which indicated that VitC and RP/HPR cotreatment had a stronger effect on resisting UV‐induced senescence. Previous studies have found that the major facets of senescence included two pathways responsible for establishing and maintaining a senescence program, P21 and P16 have vital role in these pathways [24]. In our study, additional mechanistic studies (Figures 6, 7, 8, 9) revealed that a combination of VitC and RP/HPR could significantly inhibit expression of the senescence‐related genes P21 and P16 by activating CRABP2 expression, inhibit the SASP release, slow‐down elastin degradation, and promote the gene expression of COL‐I and COL‐IV to delay the senescence of skin induced by UV irradiation.

The innovation of RP/HPR and VitC formulations is that their stability and permeability have been improved. Vitamin A and VitC can be oxidized readily and lose activity in a conventional formulation. The derivatives of RP/HPR and VitC have higher stability and can be active in a product for a long time [6, 8]. In addition, the molecular structure of these two ingredients has been improved to enable penetration into the deep layers of skin [1].

RP/HPR and VitC also have advantages in terms of safety [1, 4, 8]. These ingredients are natural antiaging and skin‐whitening substances that are safe if formulations have been created appropriately and the method of use is correct. However, due to individual differences, sensitive skin may be allergic to these ingredients. Therefore, before use, it is recommended to conduct a skin test to ensure safety.

## 5 Conclusions

A combination of VitC and RP/HPR at a weight ratio of 40:3 was shown to be the optimal combination for the antiaging, luster, and whitening of skin. Their joint action directly inhibited expression of the senescence‐related genes P21/P16 induced by UV irradiation by activating CRABP2 expression, inhibiting the SASP release induced by UV irradiation, significantly slowing down elastin degradation, and promoting the gene expression of COL‐I and COL‐IV so as to delay the senescence of skin induced by UV irradiation. Compared with RP/HPR monotherapy or VitC monotherapy, a combination of VitC and RP/HPR was more efficacious in terms of promoting collagen regeneration and preventing skin aging, which could be a potent and safe strategy to prevent skin aging and improve skin whitening.

## Author Contributions

All authors made a significant contribution to the work reported, whether that is in the conception, study design, execution, acquisition of data, analysis, and interpretation, or all these areas; took part in drafting, revising, or critically reviewing the article.

## Conflicts of Interest

The authors declare no conflicts of interest.

## Supporting information


Appendix S1.

Appendix S2.

## Data Availability

The data are available from the corresponding authors upon reasonable request.
