# Supplementary material for: A Functional Skincare Formulation Mixed With Retinyl Propionate, Hydroxypinacolone Retinoate, and Vitamin C on Antiaging and Whitening Han Women in Shanghai, China
Source: J Cosmet Dermatol. 2025 Mar 3;24(3):e16747. doi: 10.1111/jocd.16747 (PMC11875225; doi:10.1111/jocd.16747)
Supplement: Supplementary file 1 — Appendix S1. [file JOCD-24-e16747-s002.docx]

**The inclusive and exclusive criteria of human volunteer (HV) study**

Inclusive criteria

1. Healthy women of 25~ 40 years with obvious fine wrinkles around the corners of their eyes.
2. Can cooperate with the investigator and maintain a regular lifestyle during the trial.
3. Be able to read and understand the contents of the informed consent form (ICF), and voluntarily sign the ICF.
4. Agree not to use any cosmetics, drugs, or health products that may affect the results during the trial.

Exclusive criteria

1. Having used the following products or drugs within the specified timeframe before baseline: 1 week for antihistamines, 1 month for immunosuppressants, 2 months for the local anti-inflammatory drugs at the testing site, 3 months for topical retinoid, α-hydroxy acid, salicylic acid, and hydroquinone, 6 months for prescription drugs (antibiotics, retinoid, α-hydroxy acid and steroids), oral contraceptives (if you have been taking the same kind of contraceptives for the past 6 months, you can continue to take them).
2. Individuals with a history or current diagnosis of following disease:
3. skin diseases (such as psoriasis, eczema, psoriasis, skin cancer, etc.).
4. current insulin dependent diabetes.
5. asthma or other chronic respiratory disease currently undergoing treatment.
6. those who have received anticancer chemotherapy within the past 6 months.
7. immune deficiency or autoimmune diseases;
8. bilateral mastectomy and bilateral axillary lymph node resection.
9. any other health issues or chronic diseases.
10. Breastfeeding or pregnant women.
11. Individuals whose testing site has scars, pigmentation, atrophy, nevus flammeus, uneven skin color, folliculitis, or other conditions that may interfere with the judgement of results.
12. Other situations that may affect the judgement of results by the investigator.
