# Supplementary material for: A Functional Skincare Formulation Mixed With Retinyl Propionate, Hydroxypinacolone Retinoate, and Vitamin C on Antiaging and Whitening Han Women in Shanghai, China
Source: J Cosmet Dermatol. 2025 Mar 3;24(3):e16747. doi: 10.1111/jocd.16747 (PMC11875225; doi:10.1111/jocd.16747)
Supplement: Supplementary file 2 — Appendix S2. [file JOCD-24-e16747-s001.docx]

Compared with the control group, after using HPR/VitC samples for 14 and 28 days, the improvement rate of fine lines area was 9.15% and 14.71% respectively, and the difference was significant. When using the same time (14 days and 28 days), the improvement rate of fine lines area in group D was higher than that in the other three groups.

Compared with the control group, after using HPR/VitC samples for 14 and 28 days, the improvement rate of crow's feet was 10.89% and 14.49% respectively, and the difference was significant. When using the same time (14 days and 28 days), the improvement rate of fine lines area in group D was higher than that in the other three groups.

Compared with the control group, after using HPR/VitC samples for 14 and 28 days, the improvement rate of L* value was 1.14% and 1.59% respectively, and the difference was significant. Besides，after using HPR/VitC samples for 28 days, the improvement rate of L* in group D was higher than that in the other three groups.

Compared with the control group, after using HPR/VitC samples for 14 and 28 days, the improvement rate of a* value was 1.53% and 4.23% respectively, and the difference was significant. When using the same time (14 days and 28 days), the improvement rate of fine lines area in group D was higher than that in the other three groups.

Compared with the control group, after using HPR/VitC samples for 14 and 28 days, the improvement rate of b* value was 1.56% and 2.39% respectively, and the difference was significant. When using the same time (14 days and 28 days), the improvement rate of fine lines area in group D was higher than that in the other three groups.

Compared with the control group, after using HPR/VitC samples for 14 and 28 days, the improvement rate of ITA°value was 3.61% and 4.90% respectively, and the difference was significant. When using the same time (14 days and 28 days), the improvement rate of fine lines area in group D was higher than that in the other three groups.

Compared with the control group, after using HPR/VitC samples for 14 and 28 days, the improvement rate of the number of nasolabial folds was 13.25% and 17.09% respectively, and the difference was significant. When using the same time (14 days and 28 days), the improvement rate of the number of dharma patterns in group D was higher than that in the other three groups.

Compared with the control group, after using HPR/VitC samples for 14 and 28 days, the improvement rate of the area of nasolabial folds was 15.39% and 21.30% respectively, and the difference was significant. When using the same time (14 days and 28 days), the improvement rate of the number of dharma patterns in group D was higher than that in the other three groups.

Compared with the control group, after using HPR/VitC samples for 14 and 28 days, the improvement rate of the volume of nasolabial folds was 18.66% and 23.02% respectively, and the difference was significant. When using the same time (14 days and 28 days), the improvement rate of the number of dharma patterns in group D was higher than that in the other three groups.

Compared with the control group, after using HPR/VitC samples for 14 and 28 days, the improvement rates of R2 values were 9.93% and 13.80% respectively, and the differences were significant. When using the same time (14 days and 28 days), the improvement rates of R2 values in group D were higher than those in the other three groups.

Compared with the control group, after using HPR/VitC samples for 14 and 28 days, the improvement rates of F4 values were 10.81% and 15.10% respectively, and the differences were significant. When using the same time (14 days and 28 days), the improvement rates of F4 values in group D were higher than those in the other three groups.

Compared with the control group, after using HPR/VitC samples for 14 and 28 days, the improvement rates of fine lines were 9.92% and 12.40% respectively, and the difference was significant. When using the same time (14 days and 28 days), the improvement rate of fine lines in group D was higher than that in the other three groups.

Compared with the control group, after using HPR/VitC samples for 14 and 28 days, the improvement rates of crow's feet were 8.51% and 15.96% respectively, and the difference was significant. When using the same time (14 days and 28 days), the improvement rate of fine lines in group D was higher than that in the other three groups.

Compared with the control group, after using HPR/VitC samples for 14 and 28 days, the improvement rates of nasolabial folds were 4.55% and 9.09% respectively, and the difference was significant. When using the same time (14 days and 28 days), the improvement rate of fine lines in group D was higher than that in the other three groups.
